# Anticancer effects and lysosomal acidification in A549 cells by Astaxanthin from Haematococcus lacustris

**DOI:** 10.6026/97320630016965

**Published:** 2020-11-30

**Authors:** K Ramamoorthy, Subramanian Raghunandhakumar, RS Anand, A Paramasivam, S Kamaraj, S Nagaraj, Devaraj Ezhilarasan, Thangavelu Lakshmi, Kamal Dua, Dinesh Kumar Chellappan, Ashokkumar Veeramuthu

**Affiliations:** 1 Centre for Advanced Studies in Botany, University of Madras (Guindy Campus), Chennai, India-600 025; 2 Department of Pharmacology, Saveetha Dental College, Saveetha University, Chennai, India; 3 Centre for Biotechnology, Anna University, Chennai - 600 025; 4 Biomedical Research Unit and Laboratory Animal Centre-Dental Research Cell, Saveetha Dental College, Saveetha University, Chennai, India; 5 Department of Biotechnology, Periyar University (PG Extension Centre), Dharmapuri - 636701; 6 Discipline of Pharmacy, Graduate School of Health, University of Technology Sydney, Ultimo, NSW, 2007, Australia; 7 School of Biomedical Sciences and Pharmacy, University of Newcastle, Newcastle NSW 2308, Australia; 8 Department of Life sciences, School of Pharmacy, International Medical University, Bukit Jalil ,57000,Kualalumpur, Malaysia; 9 Department of Chemical Technology, Chulalongkorn University, Bangkok,Thailand

**Keywords:** Astaxanthin, Haematococcus lacustris, A549 cells, Apoptosis, Lysosomal acidification

## Abstract

Astaxanthin (AXN) is known to have health benefits by epidemiological studies. Therefore, it is of interest to assess the effect of AXN (derived from indigenous unicellular green alga Haematococcus lacustris) to modulate cell cycle arrest, lysosomal acidification and eventually apoptosis using in vitro in A549 lung cancer cells. Natural extracts of astaxanthin were obtained by standardized methods as reported earlier and characterized by standard HPLC and MS. Treatment of A549 cells with AXN (purified fraction) showed significant reduction in cell viability (about 50%) as compared to crude extract at 50µM concentration. Thus, we show the anticancer effects and lysosomal acidification in A549 cells by Astaxanthin from Haematococcus lacustris for further consideration. Together, our results demonstrated the anticancer potential of AXN from Haematococcus lacustris, which is found to be mediated via its ability to induce cell cycle arrest, lysosomal acidification and apoptotic induction.

## Background

Haematococcus lacustris (formerly called as Haematococcus pulvialis) is a freshwater microgreen alga with intirinsic antioxidant property and is reported to confer numerous health benefits [1,2]. Astaxanthin (AXN) (3, 3'-dihydroxy-β, -β, carotene 4,4'-dione) is a pigmented carotenoid antioxidant with high biological activity given its potential in food and pharmaceutical industries, as well as in feed industry for the ability to pigment and improve survival rate of aquatic animals. H. lacustris has been used preferably to produce astaxanthin from natural sources, and is known to possess high content of astaxanthin at about 5%, of its dry weight (DW) [3]. Furthermore, the structure of astaxanthin from H. lacustris is similar to those in salmon and other aquatic organisms, making astaxanthin more easily to be absorbed. As a result, many efforts have been focused on production of astaxanthin-rich H. lacustris to exploit its benefits to promote human health and as well in industrial applications [4]. Astaxanthin (AXN) from various microalgae and yeast has been approved by United States FDA as a neutraceutical and also as a nutraceutical supplement for about 15 years [5,6]. Studies using various in vitro and animal models documented significant health benefits including mediating immune response [7], inhibiting growth of colorectal cancer cells [8], improving rodent memory functions [9] and protection against UV rays mediated photooxidation, antioxidant and anti-inflammatory functions [2]. In addition, reports suggest that the unique structural properties of AXN endure it to span cell membrane and quenches free radicals thereby serving as a better pharmaceutical agent [10,11]. Notably, natural AXN has been shown to cross blood brain barrier (BBB) and exerts protections against various neurological disorders including Alzheimers Disease (AD) [12] and inhibits growth of various tumors including fibrosarcoma, breast, and prostate cancer cells [8,13]. However, most of the reported correlated the beneficial effects of natural AXN for its antioxidant activity and the mechanistic insight underlying natural AXN remains largely unknown. Therefore, it is of interest to assess the effect of AXN (derived from indigenous unicellular green alga Haematococcus lacustris) to modulate cell cycle arrest, lysosomal acidification and eventually apoptosis using in vitro in A549 lung cancer cells.

## Materials and Methods:

### Chemicals and Reagents:

Propidium iodide (PI), Acridine orange (AO), Ethidium bromide (ETBR) and Dimethyl sulfoxide (DMSO), 3-[4, 5-Dimethyl-2-thiazolyl]- 2,5-diphenyl tetrazolium bromide (MTT) and Acridine orange (AO), ethidium bromide (ETBR) were purchased from Sigma (St. Louis, MO, USA). PMI 1640 medium, fetal bovine serum (FBS) was purchased from Gibco (Carlsbad, CA, USA). FBS was stored at -20°C and then freshly prepared aliquots were used for cell culture complete medium. All the other chemicals used were of analytical grade unless otherwise stated.

### Extraction of Astaxanthin:

The two litre of 30 days old culture of H. lacustris grown in BBM medium was taken and centrifuged at 10,000 rpm for 15 minutes. After centrifugation, the supernatant was decanted and the pellets were homogenized in a mortar and pestle using 100% acetone in the ratio of 1:10 v/v and then centrifuged at 8,000 rpm for 10 minutes. The supernatant containing pigments was retained and the pellet was again homogenized in fresh solvent and subsequently centrifuged until the cell pellets became colorless. The combined extracts were concentrated with rotary evaporator at 60°C. The dry solid residue was re-dissolved in acetone for subsequent separation such as TLC and column chromatography technique and the different elutes of the sample were confirmed by spectral and analytical methods with authentic astaxanthin.

### HPLC analysis: 

The samples were analyzed using LC20AD HPLC (SHIMADZU, Japan) equipped with binary pump, auto sampler and fully automated computer-controlled system with Lab solution chromatography workstation for data analysis. Astaxanthin was separated by chromatographic column inert sustain swift C-18, 4.6mmx150mm- 3µm, (GL sciences Inc.). 20µl volume was injected with 1ml/min flow rate at 25°C column temperature under 474nm wavelength with mobile phase consists of acetonitrile/methanol/dichloromethane (80:15:5, v/v/v).

### MALDI-LC/MS analysis:

Mass spectra using MALDI-LC/MS ((Bruker Daltonics) with spectrometer, equipped with a nitrogen laser (l = 337 nm), operated in reflectron, positive ion mode. a-cyano-4-hydroxy-cinnamic acid used as the matrices with three independent sample spotting along with Astaxanthin standard samples in ratio of 1:1 (0.5µl of sample: 0.5µl of matrix) were spotted on stainless steel MALDI-LC/MS 324 well plate from Bruker Daltonics.

### Cell line and culture:

Human lung cancer cell line A549 were purchased from National centre for cell science (NCCS), Pune. Cells were maintained in Dulbecco's modified Eagle's medium (DMEM) supplemented with 10% fetal bovine serum (FBS) and 1% Antibiotic-Penicillin streptomycin solution. Cells were maintained at a controlled condition of 5% CO_2_, and 95% humidity. Experiments were performed in 6-well plates and 96 well plates unless stated otherwise. Cells were seeded at a density of 1 x 106 and 1 x 104 cells per well and incubated for 24 h prior to the experiments. The cells were washed with (phosphate buffered saline, pH 7.4) PBS and incubated in fresh medium containing different concentrations of AXN.

### Cytotoxicity assay:

Effect of AXN (dose dependent) on cell viability was measured using the 3-(4,5-dimethylthiazol-2-yl)-2,5- diphenyltetrazolium bromide dye reduction was performed according to our pervious procedure [14]. Finally, the concentrations of AXN resulting in 50% reduction in cell viability at 24h (i.e., 50µM/ml) were then calculated and were used for further experiments. Results were expressed as mean of three independent experiments.

### Acridine orange Ethidium bromide staining:

AO/EB double staining was performed using standardized methods as previously reported [15]. A549 cells from all the experimental groups were stained using AO (4 µg/ml) and ETBR (4 µg/ml) for 15 min. Followed by incubation-stained cells were examined under the fluorescence microscope using red and green filters. Viable cells will exhibit uniform bright green nuclei denoting normal structure, whereas early apoptotic cells will exhibit green nuclei, but will be more condensed. Late apoptotic cells will show orange to red nuclei with condensed chromatin, while the necrotic cells will show orange to red nuclei with basal chromatin levels.

### Statistical analysis:

All the experiments were performed independently as triplicates (n=3). The mean ± standard deviation (SD) was determined for each group. One-way analysis of variance (one-way ANOVA) and Tukeys' test was used for statistical analysis. Statistical value of p <0.05 was used for considering statistical difference between groups.

## Results:

### Identification and Characterization of AXN:

**Figure 1:**
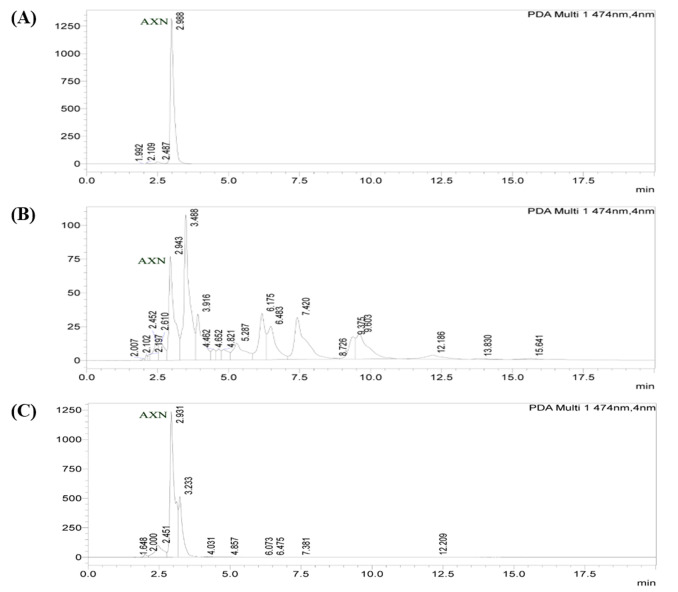
The HPLC analysis of astaxanthin extracted samples: crude (B), purified (C) from H. lacustris and standard astaxanthin substance (A) with respective of three independent experiments.

**Figure 2:**
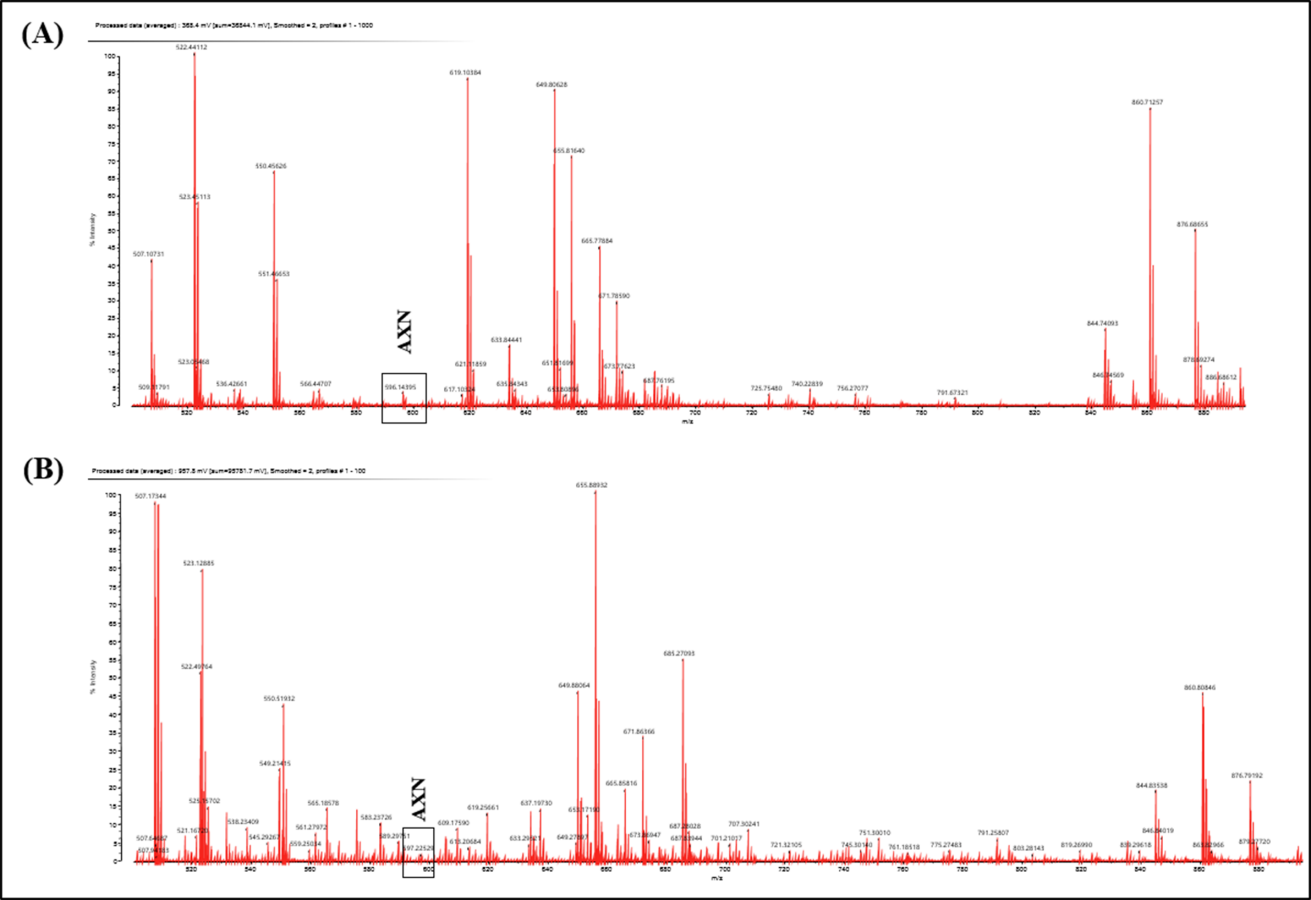
MALDI-LC/MS spectra with matrices of (a) unknown compound isolated and purified from H. lacustris and (b)standard AXN substance were observed in LC/MS. A peak at m/z at 596 ∼ 597 has shown from both unknown and known standard of AXN.

The extracted AXN (Crude & Purified) from H. lacustris was identified, characterized, validated and finally confirmed with MALDI-LC/MS and HPLC analysis. The structure and molecular weight based characterization and determination of sample content between crude and purified AXN was clearly mentioned in Figure 1 & Figure 2. In order to characterization of AXN, the levels of stability, linearity, precision and reproducibility and refractory period of experiments were followed by standard analytical laboratory procedure.

### Cytotoxicity assay:

**Figure 3:**
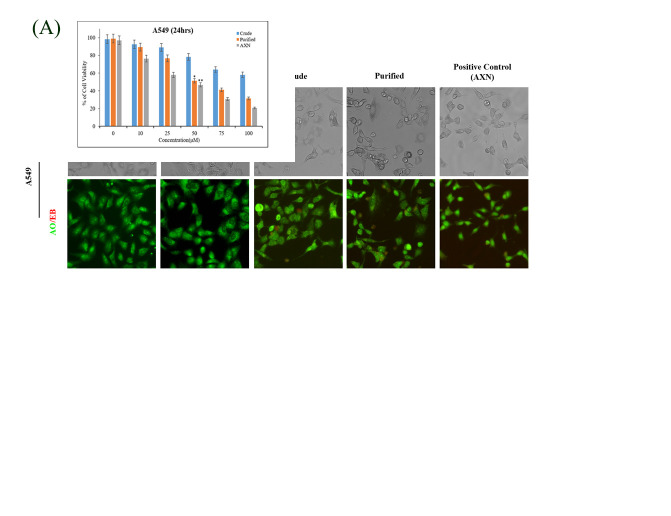
Shows the cell viability analysis by MTT assay. A549 cells were seeded onto each well and allowed to grow overnight. Cells were treated with various concentration of AXN (crude, Purified and standard AXN) for 24 h and the inhibitory concentration (IC50) was determined. Experiments were performed in triplicates, representative images were presented here and the results were expressed as mean ± SD of 3 experiments. *Denotes significant difference from control by student's t-test (p < 0.01). (B) Shows status of lysosomal acidification (via pore formation and cytotoxicity) in A549 cells. Cells were treated with and without AXN 50 µM (crude, Purified and standard AXN) for 24 h. Control and AXN treated cells were stained with AO/ETBR and the dual staining was examined under fluorescence microscopy (AO: excitation wavelength: 490nm and emission wavelength: 640nm). (ETBR excitation wavelength: 520nm and emission wavelength: 600nm). Presence of bright red fluorescence indicates lysosomal acidification. Results are expressed as mean ± SD for 3 independent experiments. *denotes significant difference from control by student's t-test (p < 0.01). Magnification: X20, Scale bar: 50µM.

Treatment of crude and AXN purified fractions from H. lacustris induced significant (P<0.05) cell death in A549 lung cancer cells. Cell death was observed within 24 hours of treatment with the crude and purified fractions of AXN, while control AXN (standard) was also producing similar cell death at 50 µM treatment concentrations in both the cell lines studied (Figure 3A).

### AXN induces nuclear morphology changes by lysosomal acidification:

Considering that AXN induces a G2/M phase arrest blocking the progression of cell growth and replication, it is obvious to expect deregulated orchestration in the intracellular organelle functions including lysosomes. Indeed, Lysosomes are reported to play important role in all forms of cell deaths (apoptosis, autophagy and necrosis) associated with cancer [16]. We evaluated the changes in lysosomal acidification using acridine orange staining in A549 cells treated with crude and purified AXN fraction from H. lacustris and compared it with control and pure AXN positive control. The staining revealed that both purified AXN fraction and positive controls induced significant (P< 0.05) lysosomal acidification as observed by the increase in red intensity within the cells (Figure 3B).

## Discussion:

Cancer mediated death in global population remains at top for several decades while there is continuous approval for array of anticancer drugs by FDA. About 59 new drugs in 2018 and 46 new drugs in 2017 were approved by FDA, denotes the need for new therapies that can reduce oncology-associated pathologies [17]. Though there are several synthetic drugs that hit global market for oncology therapies, imputability of adverse reactions of the same remains inevitable [18]. Hence, the avenue for safer anticancer drugs with minimal adverse reactions remains open and the agents derived from natural sources possess significant benefits as evident from accumulating reports [19,20]. AXN from H. lacustris has been known to possess diverse biological functions including anticancer actions. Other studies using AXN from natural sources already documented its anticancer effects against breast [21], Oral [22], bladder [23], colon [24] and liver [25] however its underlying mechanisms remain to be elucidated. Here we have attempted to investigate the effect of AXN on cell cycle arrest and cellular organelle dysfunction which can be attributed to its anti cancer activity. Although, many carotenoids have been reported to exert anti cancer and antiproliferative effects, its potential use at clinical stages need to be validated. This could be due to the fact that studies so far conducted failed to establish how AXN affects cellular process or to establish a single prime mode of its anti-cancer action. In this study AXN treatment altered viability of A549-lungcancer cells. Viability was reduced upto 50% at 50µM concentration of purified AXN. However, time kinetics of purified AXN did not show any changes in loss of viability after 24 hrs (Data not shown).

Studies using other plant-derived carotenoids have shown they inhibit cell cycle progression and mediate cell cycle arrest, particularly in the G0/G1 phase of cell cycle [26,27]. Our results showed that AXN treatment to A549 cells caused accumulation of cells in G0/G1 phase, concomitantly reducing the total percentage of cells in G2/M phase as well. Considering the similarities with other studies, it is found that AXN ability to mediate cell viability loss is associated with cell cycle arrest and inhibition of cell proliferation. Similar to our results many studies have correlates the antitumor efficacy of carotenoids for its ability to mediate cell cycle arrest [28, 29]. This suggest that cell cycle arrest might be a initial mode of mechanism of antitumor action of AXN as reported in other cancer cells like colon and prostate cells [29,30]. Further to elucidate the phenomenon that lies behind AXNs ability to mediate cell cycle arrest, we have analyzed the status of cellular organelle dysfunction in A549 cells upon AXN treatment. Indeed, lysosomes are critically noted as a crucial cellular organelle during treatment with various anticancer drugs, as it contains numerous hydrases that can be to degrade intra and extracellular materials and is implicated in cell death [31,32]. Notably, involvement of lysososmes in cell death has been overlooked because lysosomal membrane permeabilization (LMP) due to lysosomal acidification does not alter its ultrastructure [33]. Perhaps, studies involving LMP has been recently focused due to development of more assays that analyze LMPs. Various stimuli such as lysosomotrophic detergents, viral proteins, bacterial and fungal toxins, proteases, lipid and lipid metabolites, and notably ROS were known to modulate LMP thereby causing lysosomal acidification and eventually cell death [34]. In this study, treatment with AXN to A549 cells causes significant increase in acridine orange (AO) staining denotes increased number of acidic autolysosomes as revealed by Fluorescent microscopic analysis. This denotes that AXN mediates lysosomal acidification and thereby causing loss of LMP and releasing cath D hydrolytic enzymes that can act along with caspases to mediate apoptotic cell death. The observed increase in lysosome acidification can be attributed to ability of AXN to generate ROS in cancer cells and can be correlated to its anticancer function. Here we are the first to report AXN induces lysosomal acidification in A549 cells and can be prime mechanism by which it exerts anti cancer role.

## Conclusion

We show the anticancer effects and lysosomal acidification in A549 cells by Astaxanthin from Haematococcus lacustris for further consideration.
